# Battery for fall risk assessment in older adult people—BARQ: analysis of reliability and objectivity

**DOI:** 10.3389/fpubh.2024.1456564

**Published:** 2025-01-29

**Authors:** Andrea Carmen Guimarães, Brisa D’Louar Costa Maia, Karollyni Bastos Andrade Dantas, Gustavo Costa Santos, Letícia Moreira Américo, Nelzy Aparecida Silva Werner, Lucio Flávio Gomes Ribeiro da Costa, César Augusto da Silva Santos, Estélio Henrique Martin Dantas

**Affiliations:** ^1^Department of Medicine, Program in Health Sciences, Federal University of Lavras, Lavras, Brazil; ^2^Department of Medicine, Doctor of Federal University of São João del-Rei, São João del-Rei, Brazil; ^3^Master’s and Doctoral Program in Biosciences and Health - PBS, Program in Health and Environment - PSA, Tiradentes University - UNIT, Aracaju, Brazil; ^4^Department of Medicine, Faculty of Health Sciences, Federal University of Lavras, Lavras, Brazil; ^5^Doctor of State University of Pará, Belém, Brazil; ^6^Program in Nursing and Biosciences - PPgEnfBio, Federal University of the State of Rio de Janeiro, Rio de Janeiro, Brazil

**Keywords:** accidental falls, visual acuity, gait analysis, risk factors, proprioception, reproducibility of results

## Abstract

**Introduction:**

This study aims to address significant gaps in fall risk assessment among older adult individuals, using a methodological approach to develop and validate effective instruments.

**Objective:**

To establish the objectivity and reliability of the Battery for Fall Risk Assessment in older adult people - BARQ.

**Methodology:**

This descriptive, cross-sectional, exploratory study started with a preliminary version of BARQ, based on the Comprehensive Falls Risk Screening Instrument—CFRSI. The following variables were included: Fall History and Adverse Events (FH), Medication Use (MU), Home Safety (HS), Balance (Ba), Mobility (Mo), and Visual Acuity (VA). BARQ was administered to 136 older adult participants (X̅ = 70.3 ± 7.20 years) by two assessment teams (∝ and *β*), at three different times, to examine instrument reliability and objectivity. Statistical analyses included Pearson’s correlation, Cronbach’s alpha coefficient, Student’s *t*-test, and Hedges’ g.

**Results:**

Intra-rater and inter-rater correlations were significant for mobility (*r* = 0.90 and *r* = 0.88, respectively; *p* < 0.01) and visual acuity (*r* = 0.86 and *r* = 0.87, respectively; *p* < 0.01). Cronbach’s alpha coefficients indicated nearly perfect reliability for mobility (0.97) and visual acuity (0.96). No statistically significant differences were found in intra-rater assessments (p ranging from 0.11 to 0.55) and inter-rater assessments (p ranging from 0.37 to 0.55). Hedges’ g test showed medium effect sizes for the overall fall risk index between S1 and S2 (*g* = 0.39) and small to medium for other variables.

## 1 Introduction

Alongside cardiovascular, gastrointestinal, neurological, emotional, and respiratory diseases, falls among older adult individuals represent one of the primary health hazards leading to hospitalizations in this population GBD (1). Several factors increase the risk of falls in older adult people, such as advanced age, low educational attainment, polypharmacy, malnutrition, living alone, urban residency, smoking, and alcohol consumption (2). Moreover, conditions like heart disease, hypertension, diabetes, stroke, frailty, history of falls, depression, Parkinson’s disease, and pain also contribute to higher incidence of falls among older adult individuals (2). Conversely, higher education has been identified as a protective factor against falls among the older adult (2). According to Jaul and Barron (27), aging is a universal but non-uniform process. Awareness of age-related physiological changes such as reduced visual and auditory acuity, slower reaction times, and impaired balance can prepare patients and caregivers to manage risks, make informed decisions, and possibly prevent falls and medication-related adverse effects (27). According to Xing et al. (28), the research on identifying the pathogenic factors of falls caused by balance disorders in the older adult is a premise and foundation for identifying, evaluating, and controlling dysfunctions and loss of independence. From 2000 to (1), falls in the gerontological group accounted for 60.4% of hospitalizations (3), highlighting a significant public health issue and substantial healthcare costs in this area. Given these data, the study of falls among older adult people assumes increasing importance proportional to the progressive growth of this population. Among the older adult, falls not only cause injuries but also fatalities. Globally, nearly 37.3 million falls receive medical treatment in hospitals each year. Additionally, there are 646,000 catastrophic falls resulting in death, with individuals over 65 years old experiencing the majority (4). Approximately 30% of older adults experience at least one fall annually, a trend expected to increase to over 40% among individuals over 70 years old (5). It has been reported that about 25% of older adult patients who experience a fall will have at least one more fall event within 6 months (6).

One type of fall with a higher incidence is Falls from Standing Height (FSH), representing 67.1% of the total reported (7). There are various types of FSH, which can be classified according to the cause, mechanics, or consequence of the fall: lateral falls onto the arm or elbow, where the older adult fall sideways, can occur due to loss of balance or tripping. In a fall onto the arm, the impact is absorbed by the arm and shoulder, whereas in a fall onto the elbow, the impact is absorbed by the elbow and forearm; forward falls, where the older adult fall forward, can occur due to tripping, imbalance, or vision problems; backward falls, where the older adult fall backward, can occur due to dizziness, fainting, or neurological problems; falls from a lying position, where the older adult fall from a bed or chair to the floor, can occur due to mobility issues, instability when getting up, or inattention; and multidirectional falls, where the older adult fall without a defined direction, can occur due to a sudden loss of balance.

The causes of falls, and consequently their risk factors, are of intrinsic nature (age, gender, functional capacity, chronic diseases, gait disorders, prior history of falls, or psychocognitive dysfunctions), according to Minta et al. (8), or extrinsic nature (environment and medications used), according to Poh and Shorey (9). Therefore, instruments to assess the risk of falls in older adult individuals need to be multifactorial to encompass all involved variables.

The starting point for establishing a methodology to assess the risk of falls in older adult individuals originated from the study by Fabre et al. (10), which established an algorithm to identify variables present in previously published studies. Based on this study, the Fall Risk Assessment Battery for older adult Individuals (BARQ) was developed, which is the focus of the present article.

Considering the above, this article aims to establish the objectivity and reliability of the BARQ.

## 2 Methodology

This study was structured as a descriptive, cross-sectional, and exploratory study, aiming to explore areas with limited prior information or issues with limited scientific evidence (11). Additionally, a methodological approach was adopted to explore scientific methods involving the creation, validation, and evaluation of instruments (12), using the Delphi technique to ensure the scientific validity of the instrument (13).

The initial phase of the study involved the development of a preliminary version of instruments to assess the components of fall risk in the older adult, based on the Comprehensive Falls Risk Screening Instrument (CFRSI) (10). This stage included a literature review in databases such as Web of Science, Scopus, SciELO, EMBASE, Cochrane Library, LILACS, and PubMed, using descriptors: Falls risk assessment, Geriatric assessment, Balance assessment, Gait analysis, and Fall prevention. The results were analyzed using the Brainstorming technique (14), conducted by researchers from the Laboratory of Human Motor Science—LABIMH, culminating in the Fall Risk Assessment Battery for older adult Individuals (BARQ).

It was decided that the BARQ should initially cover the following variables: History of Falls and Injuries (HFI), Medication Use (MU), Home Safety (HS), Balance (Ba), Mobility (Mo), and Visual Acuity (VA). For the first three variables, the CFRSI questionnaires were adapted and supplemented.

The HFI included nine questions addressing: occurrence of falls in the past 3 years, specifying if any occurred in the last 12 months; difficulties walking; presence of arthritis; drug reactions; purchasing medications from the same pharmacy; need for corrective lenses and ophthalmological follow-up.

The MU, composed of four questions, examined: whether the individual takes prescribed medications; occurrence of side effects; description of these effects; the quantity and types of medications used. Participants were asked to bring the medication packages to ensure the accuracy of the responses. Medications were categorized by trade name, active substance, and medicinal effect, with the classification done by a pharmacist and a physician.

Finally, the HS, with 12 questions, aimed to assess home safety for the older adult, identifying potential risks of falls and accidents. The questions included aspects such as the presence of handrails on stairs, adequate lighting, grab bars in the bathroom, non-slip mats, and the organization of the environment.

The variables Balance, Mobility, and Visual Acuity were assessed using established classical tests, namely: Functional Reach Test (15), Expanded Timed Up-and-Go (ETUG) (16), and the Snellen Chart (17), respectively.

The full formulation of the BARQ can be seen at: https://abrir.link/MLdHn.

Based on the consideration of the raw values and their geometric mean, the formula for the General Fall Risk Index (GFRI) was derived, also available at the aforementioned link.

To evaluate the reliability (intra-rater error, using the test–retest method) and objectivity (inter-rater error, through agreement between observations) of the instrument, the BARQ was applied to the same Reference Group (RG) by two different teams of assessors: team alpha (∝) collected data in week 1 (S1) and the subsequent week, S2, to ascertain intra-rater error, assessing the reliability of the battery.

Team beta (*β*) applied the BARQ to the RG in the third week (S3) to evaluate inter-rater error, allowing the assessment of objectivity. Both teams were homogeneous, comprising three assessors each, all with master’s or doctoral degrees and experience working with older adult individuals.

The RG consisted of 136 older adult volunteers (mean age = 70.3 ± 7.20 years), all participants in community groups from the states of Sergipe and Bahia (Brazil). Participants were randomly selected after verifying inclusion criteria (being over 60 years old, volunteering for the study) and exclusion criteria (contraindication in the Revised Physical Activity Readiness Questionnaire: r-PARQ or failure to complete the three evaluation sessions).

The study was conducted in full compliance with Law n°: 14,874, of May 28, 2024 (18), and the Declaration of Helsinki (19), with all participants signing the Informed Consent Form (TCLE). The project was submitted to the ethics committee of Tiradentes University and approved under opinion n°: 6,847.94, of May 24, 2024, in the project of CAAE n°: 26524719.4.0000.5371.

To ensure the analysis of the objectivity and reliability of the BARQ, a thorough statistical procedure was applied using SPSS 22.0 for Windows (IBM Corp., Armonk, NY, United States). Initially, a descriptive analysis was conducted to characterize the studied sample. Descriptive statistics, such as means, standard deviations (SD), and frequencies, provided an overview of the collected data, allowing an initial understanding of the sample’s main characteristics.

To evaluate the association between measurements obtained by the same assessors and by different assessors, Pearson’s test was used. The internal consistency of the measurements was verified using Cronbach’s alpha. To assess differences in intra-rater and inter-rater measurements, Student’s *t*-test was applied. Additionally, Hedges’ g test was employed to verify the effect size in measurements performed by the same and different assessors.

A significance level of *p* < 0.05 was adopted for all statistical inferences. This standard is commonly accepted in scientific research and indicates a 5% probability that the observed results are due to chance, providing a high degree of confidence in the study’s findings.

## 3 Results

The presentation of the results will begin with the characteristics of the Reference Group (RG), as shown in Table 1.

**Table 1:** General characteristics of the reference group.

Variable	Minimum	Maximum	Mean ± SD
Age	60.0	87.0	70.3 ± 7.20
MU	−4.0	1.5	−0.9 ± 1.88
FH	−6.0	6.0	2.4 ± 2.98
SD	−4.0	10.0	2.9 ± 3.29

Results in Table 1 demonstrate that the sample group is homogeneous in terms of age, which ranges from 60 to 87 years. However, the sample is heterogeneous concerning medication use, fall history, and home safety, as reflected by the high standard deviation. This variability is explained by the significant differences in medication use and the number of falls in the past year.

Table 2 shows the descriptive characteristics of the data obtained in the three assessments to determine reliability (intra-rater error, using the test–retest method) and objectivity (inter-rater error, through agreement between observations) for the three tests performed on the general index calculated. Except for the GFRI, it can be observed that the evaluations in intra-rater measurements were higher than in inter-rater evaluations.

**Table 2:** Descriptive characteristics of the data obtained in the sample at three evaluation moments.

Variable	Minimum	Maximum	Mean ± SD
Ba - 1:1	0.0	38.0	20.5 ± 7.19
Ba - 1:2	−4.0	1.5	- 0.9 ± 1.88
Ba - 2:1	−6.0	6.0	2.4 ± 2.98
Mo – 1:1	11.3	26.5	16.7 ± 3.66
Mo – 1:2	7.6	24.7	15.6 ± 3.86
Mo – 2:1	11.2	25.3	17.1 ± 3.51
VA - 1:1	0.0	11.0	9.1 ± 2.17
VA – 2:1	0.0	11.0	9.0 ± 2.18
GFRI – 1:1	12.3	25.0	17.9 ± 2.86
GFRI – 1:2	8.4	30.9	19.6 ± 5.33
GFRI – 2:1	11.4	24.6	18.5 ± 2.89

Table 3 displays the data gathered from the reference group (RG) at three evaluation points: *α*/S1, α/S2, and *β*/S3. These data were analyzed using Pearson’s correlation, and, complementarily, the Intraclass Correlation Coefficient (ICC) was calculated to provide a more comprehensive assessment of reliability and objectivity.

**Table 3:** Statistical significance of reliability (intra-rater correlation ∝/S1 x ∝/S2) and objectivity (Inter-Rater Correlation ∝/S2 x β/S3).

	*r*	*p*	*ICC*
Reliability / Intra-Rater Correlation			
Ba	0.44	0.01	0.92
Mo	0.90	< 0.01	0.88
VA	0.86	< 0.01	0.89
GFRI	0.47	< 0.01	0.92
Objectivity / Inter-Rater Correlation			
Ba	0.65	< 0.01	0,93
Mo	0.88	< 0.01	0.89
VA	0.87	< 0.01	0.90
GFRI	0.69	< 0.01	0.93

The results presented in Table 3 suggest strong reliability and consistency in both intra-rater and inter-rater evaluations, particularly in mobility and visual acuity assessments. The balance measures show varied degrees of correlation but remain statistically significant, indicating some level of consistency. The General Fall Risk Index also shows notable reliability between evaluation moments.

Table 4 presents the Cronbach’s alpha index results for the studied variables, assessing their internal reliability. This index verifies the consistency of scales and questionnaires. In this study, it is used to analyze the accuracy of mobility, balance, visual acuity, and fall risk assessments, ensuring the validity of the results obtained.

**Table 4:** Cronbach’s alpha internal reliability and consistency index for studied variables.

Variable	Alpha index	Reliability
Ba	0.75	Substantial
Mo	0.97	Almost Perfect
VA	0.96	Almost Perfect
GFRI	0.79	Substantial

Table 4 highlights the high internal reliability of the studied variables. The mobility (Mo) and visual acuity (VA) variables presented Cronbach’s alpha indexes of 0.97 and 0.96, respectively, indicating almost perfect reliability. The balance (Ba) and the General Fall Risk Index (GFRI) also showed good consistency, with substantial indexes of 0.75 and 0.79, respectively.

Table 5 presents the results of the Student’s *t*-test used to verify the statistical significance of the differences observed in both intra-rater and inter-rater evaluations. The analysis of these results will allow assessing whether the discrepancies in evaluations conducted by the same or different raters are statistically relevant, directly impacting the consistency and reliability of the obtained data.

**Table 5:** Evaluation of statistical significance of intra-rater and inter-rater differences.

Reliability - intra-rater correlation				
Variable	∝/S1	∝/S2	*t*	*p*
Ba	20.5 ± 7.19	18.6 ± 7.29	1.11	0.27
Mo	19.5 ± 3.69	18.9 ± 3.78	0.65	0.52
VA	8.5 ± 2.50	9.1 ± 2.17	−1.06	0.30
GFRI	17.9 ± 2.86	19.6 ± 5.33	−1.63	0.11

Objectivity - inter-rater correlation				
Variable	∝/S2	*β*/S3	*t*	*p*
Ba	20.5 ± 7.19	21.6 ± 7.28	−0.64	0.52
Mo	19.5 ± 3.69	18.9 ± 3.46	0.60	0.55
VA	8.5 ± 2.50	9.0 ± 2.18	−0.90	0.37
GFRI	17.9 ± 2.86	18.5 ± 2.89	−0.84	0.41

Table 5 reveals that the absence of statistically significant differences between evaluations, both intra-rater and inter-rater, indicates the homogeneity of the results. This suggests consistency and uniformity in the conducted measurements, strengthening the reliability of the evaluations and the robustness of the obtained data.

Finally, Table 6 presents the difference between measurements performed at different times and by different raters, aiming to determine the effect size using Hedges’ g test. This test was chosen because it adjusts for sample size, providing more precise estimates, especially in studies with smaller samples. Balance, mobility, visual acuity, and the general fall risk index were evaluated among the investigated variables.

**Table 6:** Effect size analysis using hedges’ g test in conducted measurements.

Variable	S1	S2	*g*	Classification
Ba	20.5 ± 7.19	18.6 ± 7.29	0.26	Medium
Mo	16.7 ± 3.66	15.6 ± 3.86	0.31	Medium
VA	8.5 ± 2.50	9.1 ± 2.17	0.25	Medium
GFRI	17.9 ± 2.86	19.6 ± 5.33	0.39	Medium

Variable	∝	β	*g*	Classification
Ba	20.5 ± 7.19	21.6 ± 7.28	0.15	Small
Mo	16.7 ± 3.66	17.1 ± 3.51	0.11	Small
VA	8.5 ± 2.50	9.0 ± 2.18	0.21	Medium
GFRI	17.9 ± 2.86	18.5 ± 2.89	0.19	Small

The Table 6 highlights that, between moments S1 and S2, the general fall risk index showed the largest medium effect size (*g* = 0.39). In the comparison between evaluators alpha and beta, visual acuity (*g* = 0.21) was the only variable with a medium effect size (Table 6). This is positive in a validation study, as small effect sizes indicate greater reliability and objectivity, demonstrating that the assessment instrument or method provides consistent and precise results over time and across different evaluators. It is important to note that there were no large effect sizes, reinforcing the reliability of the obtained results.

## 4 Discussion

The characterization of the sample, composed of 136 older adult individuals with an average age of 70.3 ± 7.20 years, revealed a demographic profile relevant for fall risk studies, such as Beretta et al. (20). The heterogeneity in terms of medication use, fall history, and home safety, reflected in high standard deviations, suggests significant variability in risk factors, corroborated by other similar studies (2, 21).

The results of the reliability and objectivity analysis of the BARQ are notable. The intra-rater correlations for mobility (*r* = 0.90, *p* < 0.01) and visual acuity (*r* = 0.86, *p* < 0.01) demonstrate high consistency of results when evaluated by the same individual at different times, values compatible with studies of a similar nature, such as Almeida Lima et al. (22) and Chardon et al. (23). Inter-rater consistency also showed significant results, with correlations of *r* = 0.88 (*p* < 0.01) for mobility and *r* = 0.87 (*p* < 0.01) for visual acuity. These results are congruent with studies by Lovie-Kitchin (17) and Spranger et al. (1), which emphasize the importance of repeatability and agreement among observers in clinical assessment.

The internal consistency analysis through Cronbach’s Alpha Index recorded high values for mobility (0.97) and visual acuity (0.96), indicating near-perfect reliability. The values for balance (0.75) and the general fall risk index (0.79) were substantial, aligning with the standards suggested by Quinn et al. (24) and validating the internal robustness of the instrument.

The absence of statistically significant differences in intra-rater (p ranging from 0.11 to 0.55) and inter-rater (p ranging from 0.37 to 0.55) evaluations suggests that BARQ maintains homogeneity in measurements. This finding aligns with existing literature where reliability tested by similar methods resulted in stable and precise data over time (25).

Effect sizes measured by Hedges’ g revealed medium indices for the general fall risk index between S1 and S2 (*g* = 0.39) and small to medium effects for other variables, indicating that BARQ has adequate sensitivity without large variations in measurements. This is a positive finding, as smaller effect sizes are indicative of greater consistency and precision (26).

The results of this study are highly relevant to gerontology and public health. The high reliability and objectivity of BARQ suggest that this instrument can be efficiently employed in both research and clinical practice. The application of BARQ can enable early detection of fall risks, facilitating preventive interventions that can significantly reduce the incidence of falls, consequently improving the quality of life and safety of the older adult.

Moreover, the use of BARQ in public health programs can inform the formulation of public policies aimed at fall prevention. Evidence-based strategies, such as the use of validated instruments, are crucial for resource optimization and the promotion of healthy and safe aging. Therefore, the findings of this study not only validate the efficacy of BARQ but also reinforce its importance as an assessment tool in the context of older adult health.

## 5 Conclusion

The detailed analysis of the data presented in this study robustly confirms the high reliability and objectivity of the Battery for Assessing the Risk of Falls in older adult People (BARQ). The consistency of intra-rater evaluations and inter-rater correlations indicates a reliable and valid tool for determining fall risk in the older adult.

The results showed significant intra-rater correlations, especially in the variables of mobility (*r* = 0.90, *p* < 0.01) and visual acuity (*r* = 0.86, *p* < 0.01), indicating high consistency and therefore reliability in evaluations conducted by the same evaluator at different times. In terms of objectivity, inter-rater correlations were also high, with mobility presenting *r* = 0.88 (*p* < 0.01) and visual acuity *r* = 0.87 (*p* < 0.01). These mathematical indicators reflect the uniformity and precision of the data obtained by BARQ.

The Cronbach’s Alpha Index demonstrated high internal reliability, with the variables of mobility (Mo) and visual acuity (VA) showing extremely high indices of 0.97 and 0.96, respectively, indicating near-perfect reliability. Balance (Ba) and the General Fall Risk Index (GFRI) also showed good consistency with substantial indices of 0.75 and 0.79, respectively. These values are clear indicators that the scales and questionnaires used in BARQ are coherent and precise.

Additionally, the results of the Student’s *t*-test did not show statistically significant differences between intra-rater evaluations (p ranging from 0.11 to 0.55) and inter-rater evaluations (p ranging from 0.37 to 0.55), suggesting that the measures are homogeneous and reliable over time and across different evaluators.

Effect sizes measured by Hedges’ g showed that between moments S1 and S2, the general fall risk index presented a medium effect size (*g* = 0.39), while in the comparison between alpha and beta evaluators, visual acuity presented *g* = 0.21. These small to medium effect sizes corroborate the stability of the results, indicating that BARQ is consistent and precise over time and between different evaluators.

The significance of these findings for gerontology is considerable, as a reliable instrument like BARQ allows for the accurate identification of fall risks in the older adult, one of the main causes of morbidity and mortality in this age group. The efficient application of BARQ can guide more effective clinical and rehabilitative interventions, improving the safety and quality of life of the older adult.

Furthermore, these results form a solid foundation for the formulation of public policies aimed at older adult health. The use of BARQ in public health programs can assist in the early detection of risks and the implementation of preventive strategies to reduce falls, resulting in less strain on healthcare systems and long-term care, and promoting healthier and safer aging for the older adult population.

Thus, the study fully achieves its primary objective by establishing the objectivity and reliability of BARQ, positioning this tool as essential for clinical practice and public health, as well as gerontological research.

## Data Availability

The datasets presented in this study can be found in online repositories. The names of the repository/repositories and accession number(s) can be found in the article/supplementary material.

## References

[1] GBD. Global, regional, and National Burden of diseases and injuries for adults 70 years and older: systematic analysis for the global burden of disease 2019 study. BMJ. (2022) 376:e068208. doi: 10.1136/bmj-2021-068208, PMID: 35273014 PMC9316948

[2] Xu Q Ou X Li J. The risk of falls among the aging population: a systematic review and meta-analysis. Front Public Health. (2022) 10:902599. doi: 10.3389/fpubh.2022.902599, PMID: 36324472 PMC9618649

[3] Lima J Quadros DV d Silva SLC d Tavares JP Pai DD. Custos das autorizações de internação hospitalar por quedas de idosos no Sistema Único de Saúde, Brasil, 2000-2020: um estudo descritivo. Epidemiol Serv Saúde. (2022) 31:e2021603. doi: 10.1590/s1679-49742022000100012, PMID: 35588512

[4] Larina VN Samkova IA Kudina EV. Falls as a problem of an aging population, a modern look at risk factors and assessment methods. Role of fear of falls in increasing their risk. The Russian. Arch Intern Med. (2021) 11:433–41. doi: 10.20514/2226-6704-2021-11-6-433-441

[5] Becher RD Brent VW Mayur LSLD Thomas G. The incidence and cumulative risk of major surgery in older persons in the United States. Ann Surg. (2023) 277:87–92. doi: 10.1097/SLA.0000000000005077, PMID: 34261884 PMC8758792

[6] Qian XX Chau PH Fong DY Ho M Woo J. Post-hospital falls among the older population: the temporal pattern in risk and healthcare burden. J Am Med Dir Assoc. (2023) 24:1478–1483.e2. doi: 10.1016/j.jamda.2023.07.014, PMID: 37591487

[7] Dornelles C Vasconcelos R de Aguiar J de Matos B Mariana AF Lidiane C. Evaluation and characteristics of falls of patients during hospitalization. Enfermería. (2021) 10:160–74. doi: 10.22235/ech.v10i2.2499

[8] Minta K Colombo G Taylor WR Schinazi VR. Differences in fall-related characteristics across cognitive disorders. Front Aging Neurosci. (2023) 15:1171306. doi: 10.3389/fnagi.2023.1171306, PMID: 37358956 PMC10289027

[9] Poh FJX Shorey S. A literature review of factors influencing injurious falls. Clin Nurs Res. (2020) 29:141–8. doi: 10.1177/1054773818802187 30227728

[10] Fabre J Ellis R Kosma M Wood RH. Falls risk factors and a compendium of falls risk screening instruments. J Geriatr Phys Ther. (2010) 33:184–97. doi: 10.1519/JPT.0b013e3181ff2a24, PMID: 21717922

[11] Al-Ababneh M. Linking ontology, epistemology and research methodology. Sci Philos. (2020) 8:75–91.

[12] Pandey P Pandey MM. Research methodology tools and techniques. Romania: Bridge Center (2021).

[13] Spranger J Homberg A Sonnberger M Niederberger M. Reporting guidelines for Delphi techniques in health sciences: a methodological review. Z Evid Fortbild Qual Gesundhwes. (2022) 172:1–11. doi: 10.1016/j.zefq.2022.04.025 35718726

[14] Brace T Nusser J. Guided brainstorming: a method for solving ergonomic issues. Prof Safety. (2021) 66:35–9.

[15] Duncan PW Weiner DK Chandler J Studenski S. Functional reach: a new clinical measure of balance. J Gerontol. (1990) 45:M192–7. doi: 10.1093/geronj/45.6.M192, PMID: 2229941

[16] Podsiadlo D Richardson S. The timed “up & go”: a test of basic functional mobility for frail elderly persons. J Am Geriatr Soc. (1991) 39:142–8. doi: 10.1111/j.1532-5415.1991.tb01616.x, PMID: 1991946

[17] Lovie-Kitchin JE. Validity and reliability of visual acuity measurements. Ophthalmic Physiol Opt. (1988) 8:363–70. doi: 10.1111/j.1475-1313.1988.tb01170.x 3253626

[18] BRASIL. Lei n° 14.874, de 28 de maio de 2024. Dispõe sobre a pesquisa com seres humanos e institui o Sistema Nacional de Ética em Pesquisa com Seres Humanos. Diário Oficial da União: seção 1, Brasília, DF, 28 maio (2024). Available at: https://legislacao.presidencia.gov.br/atos/?tipo=LEI&numero=14874&ano=2024&ato=677Izaq1ENZpWT381 (Accessed May 31, 2024).

[19] WMA. Declaration of Helsinki – ethical principles for medical research involving human subjects in Relatory of 64th WMA general assembly, Fortaleza, Brazil, October (2013). Available at: https://www.wma.net/policies-post/wma-declaration-of-helsinki-ethical-principles-for-medical-research-involving-human-subjects/ (Accessed April 19, 2024).

[20] Beretta MV De Paula TP Da Costa Rodrigues T Steemburgo T. Prolonged hospitalization and 1-year mortality are associated with sarcopenia and malnutrition in older patients with type 2 diabetes: a prospective cohort study. Diabetes Res Clin Pract. (2024) 207:111063. doi: 10.1016/j.diabres.2023.111063, PMID: 38110120

[21] Linn AJ Medlock SK Groos SS Ploegmakers KJ Seppala LJ Bosmans JE. Effects of a clinical decision support system and patient portal for preventing medication-related falls in older fallers: protocol of a cluster randomized controlled trial with embedded process and economic evaluations (ADFICE_IT). PLoS One. (2023) 18:e0289385. doi: 10.1371/journal.pone.0289385, PMID: 37751429 PMC10522018

[22] Almeida Lima CM Figueiredo Freitas de Campos S Andrade Dantas KB da Rocha Esch TR Scudese E Martins de Souza D. Reliability and objectivity of motor coordination assessments for wheelchair users. Retos. (2023) 48:701–7. doi: 10.47197/retos.v48.96178

[23] Chardon M Barbieri FA Petit P Vuillerme N. Reliability of obstacle-crossing parameters during Overground walking in young adults. Sensors. (2024) 24:3387. doi: 10.3390/s24113387, PMID: 38894176 PMC11174552

[24] Quinn L Tryposkiadis K Deeks J Vet D Henrica CW Mallett S. Interobserver variability studies in diagnostic imaging: a methodological systematic review. Br J Radiol. (2023) 96:20220972. doi: 10.1259/bjr.20220972, PMID: 37399082 PMC10392644

[25] McGarrigle L Yang Y Lasrado R Gittins M Todd C. A systematic review and meta-analysis of the measurement properties of concerns-about-falling instruments in older people and people at increased risk of falls. Age Ageing. (2023) 52:afad055. doi: 10.1093/ageing/afad055, PMID: 37211363 PMC10200549

[26] Kepes S Wang W Cortina JM. Heterogeneity in Meta-analytic effect sizes: an assessment of the current state of the literature. Organ Res Methods. (2023) 27:369–413. doi: 10.1177/10944281231169942

[27] Jaul E Barron J. Age-Related Diseases and Clinical and Public Health Implications for the 85 Years Old and Over Population. Frontiers In Public Health Frontiers In Public Health. (2017) 5:335. doi: 10.3389/fpubh.2017.00335 29312916 PMC5732407

[28] Xing L Bao Y Wang B Shi M Wei Y Huang X. Falls caused by balance disorders in the elderly with multiple systems involved: pathogenic mechanisms and treatment strategies. Frontiers In Neurology. (2023) 14:1128092. doi: 10.3389/fneur.2023.1128092 36908603 PMC9996061

